# *LbCML38* and *LbRH52,* two reference genes derived from RNA-Seq data suitable for assessing gene expression in *Lycium barbarum* L.

**DOI:** 10.1038/srep37031

**Published:** 2016-11-14

**Authors:** Lei Gong, Yajun Yang, Yuchao Chen, Jing Shi, Yuxia Song, Hongxia Zhang

**Affiliations:** 1 Ningxia Key Laboratory for Agrobiotechnology, Agricultural Bio-Technology Center, Ningxia Academy of Agriculture and Forestry Science, 590 Huanghe East Road, Yinchuan, Ningxia Hui Nationality Autonomous Region, 750002 China; 2 School of Life Sciences, Ningxia University, 489 Helanshan West Road, Yinchuan, Ningxia Hui Nationality Autonomous Region, 750021 China; 3 National Key Laboratory of Plant Molecular Genetics, Shanghai Institute of Plant Physiology and Ecology, Chinese Academy of Sciences, 300 Fenglin Road, Shanghai, 200032 China; 4 College of Agriculture, Ludong University, 186 Hongqizhong Road, Yantai, 264025 China

## Abstract

For quantitative real-time PCR (qRT-PCR) analysis, the key prerequisite that determines result accuracy is the selection of appropriate reference gene(s). Goji (*Lycium barbarum* L.) is a multi-branched shrub belonging to the Solanaceae family. To date, no systematic screening or evaluation of reference gene(s) in Goji has been performed. In this work, we identified 18 candidate reference genes from the transcriptomic sequencing data of 14 samples of Goji at different developmental stages and under drought stress condition. The expression stability of these candidate genes was rigorously analyzed using qRT-PCR and four different statistical algorithms: geNorm, BestKeeper, NormFinder and RefFinder. Two novel reference genes *LbCML38* and *LbRH52* showed the most stable expression, whereas the traditionally used reference genes such as *LbGAPDH, LbHSP90* and *LbTUB* showed unstable expression in the tested samples. Expression of a target gene *LbMYB1* was also tested and compared using optimal reference genes *LbCML38* and *LbRH52*, mediocre reference gene *LbActin7*, and poor reference gene *LbHSP90* as normalization standards, respectively. As expected, calculation of the target gene expression by normalization against *LbCML38, LbActin7* or *LbHSP90* showed significant differences. Our findings suggest that *LbCML38* and *LbRH52* can be used as reference genes for gene expression analysis in Goji.

Fluorescent quantitative real-time PCR (qRT-PCR) is a fast, accurate method for nucleic acid analysis. Unlike the standard reverse transcription polymerase chain reaction (RT-PCR), which detects the reaction product at the end, qRT-PCR detects and quantifies the amplified target nucleic acid in “real time” by measuring accumulated fluorescent signal during each cycle of polymerization. Therefore, qRT-PCR is more specific, sensitive and reproducible compared with standard RT-PCR^1^. However, reference gene is required for qRT-PCR to adjust the initial cDNA levels and transcriptional efficiency to offset the variation in nucleic acid purity and concentration during sample preparation, and to avoid the errors generated during sample treatment^2^. Previous studies demonstrated that very few reference genes were absolutely stable, but were only “relatively” stable under certain conditions in specific types of cells or tissues^3^,^4^. To date, some reference genes including those encoding actin, α- and β-tubulin, GAPDH, EF1*α* and ubiquitin have been identified. However, expression of these reference genes varied with different treatments and at different developmental stages of plants, which greatly affected the accuracy of target gene expression evaluation^1^,^5^,^6^. Thus, stable reference gene screening and evaluation are essential for functional studies of target genes.

As an importantly dietary and medicinal plant, Goji (*Lycium barbarum* L., 2n = 24) is cultivated in the northwest part of China for over 5 millennia due to its strong resistance to abiotic stresses as well as its economic value^7^. Its roots, leaves, and fruits contribute significant medicinal ingredients such as polysaccharide, betaine, carotene and anthocyanin, which function in improving immunity^8^, anti-oxidative stress^9^ and anti-tumor^10^ ability, scavenging free radicals^11^, as well as promoting sexual function^12^. Current researches on Goji are mainly limited in the isolation, extraction and development of active ingredients. Studies related to pharmaceutically active intermediate synthesis and molecular mechanisms underlying plant metabolism, development and stress resistance are still unavailable. Unlike plants from the same Solanaceae family such as tobacco, tomato, pepper and potato, the whole genome data of Goji are still not available. Previously, Liu and co-workers^13^ used *actin* as a reference gene to analyze the expression pattern of genes involved in carotene synthesis in Goji. However, the validity of results is questionable due to the lack of systematic and scientific screening of reference genes.

For plants lacking whole genome information, one of the standard approaches for reference gene identification is to clone gene homologous to the known housekeeping gene identified in other model plants. Alternatively, emerging chip or next-gen sequencing technology provides ample data, which can be used for reliable reference gene screening^6^,^14^,^15^,^16^. In *Arabidopsis*, a new approach using ATH1 chip was used to screen the super reference genes^17^. They found that a novel reference gene such as *PP2A* showed stable expression at different developmental stages and under different treatments than the classical reference gene such as *actin*. Macrae *et al*. used the spliceosome and proteasome genes from RNA-Seq data to normalize and calibrate target gene expression pattern in human cancer tissues^18^. Similarly, based on the RNA-Seq data of sika deer antlers, Liu *et al*. evaluated the stability of 16 standard reference genes and 5 expression-stable genes from the sequencing platforms of different tissues and treatments^19^.

In woody plants, screening of stably expressed reference genes using gene expression data from next-generation sequencing platform has been performed in plum^16^ (*P. salicina* cv. Lindl.) and wine grape^20^ (*Vitis vinifera*). But no such kind of research has been done in Goji. Here, the transcriptomic sequencing data from the leaves, flowers and fruits of Goji were systematically assessed, and 8 classical reference genes and 10 stably expressed transcripts with little variation among different tissues and treatments were selected as candidate reference genes. Their expression levels in different organs, at different development stages, and under drought stress condition were investigated by qRT-PCR. The stability of candidate gene expression was evaluated to select the best reference gene, and the newly selected reference gene was tested to normalize and analyze the expression level of a target gene *LbMYB1* under different conditions. Our findings provide a foundation for the functional studies of genes in Goji.

## Results

### Sequencing data analyses

From the transcriptomic sequencing of 14 sample databases, a total of 8,091,979,192 raw reads were obtained, including751,495,092 clean reads and 67,634,558,280 clean nucleotides after impurity filtration. The average Q20 value was up to 97.6%. We also found 144,250 Unigenes with a total length of 172,036,673 nt after sequence assembly. The average length of Unigene was 1193 nt, and that of N50 was1885 nt.

### Candidate reference gene selection

The mean values of raw fragments, coefficient of variance and annotation of the 18 selected candidate references were listed in Table 1. *LbTUB* displayed the maximal coefficient of variance (CV) value (1.005) of raw fragments. On the contrary, *LbEIF4A* showed the minimal CV value (0.079). The CV value variation among different reference genes suggests that these genes have different expression levels in different organs, at developmental stages and under different treatment conditions. Four classical reference genes *LbCYC, LbEIF4A, LbEF1β* and *LbUBQ* using our screening criteria (CV < 0.3, mean raw fragments > 500) were identified, suggesting the validity of our approach using raw fragment CV as preliminary screening criteria.

### qRT-PCR analyses of reference genes

C_T_ value reflects the abundance of reference gene expression. The higher the C_T_ value, the lower the expression level, and *vice versa*. The C_T_ values of the 18 reference genes ranged from 17.18 to 25.02 (Table 2). *LbHIS3* showed the lowest C_T_ value, whereas *LbSKIP* exhibited the highest C_T_ value. Compared with *LbActin7* and the newly identified reference genes *LbRH52* and *LbCML38*, reference genes *LbEF1α, LbHSP90* and *LbHIS3* manifested a higher expression upon different treatments. In addition, the dispersion level (standard deviation, SD) of C_T_ values is a schematic indicator of the stability of candidate reference gene expression in all tested samples. Among the 18 candidate reference genes, *LbPP2A* showed the lowest SD value. *LbCML38* and *LbRH52* also showed lower SD values (Table 2). In addition, PCR products of these candidate reference genes were checked on 1% agarose gel, and unique amplicons of expected length without distinct dimmers or non-specific products were observed (Supplementary Fig. 1).

### Evaluation of reference gene expression stability

In order to screen the best reference gene or gene combination in different organs, at different developmental stages, and under different treatment conditions, geNorm, NormFinder, BestKeeper and RefFinder were employed to evaluate and rank their expression stability, as shown in Tables S1, S2 and S3.

### geNorm analysis

**Figure 1:**
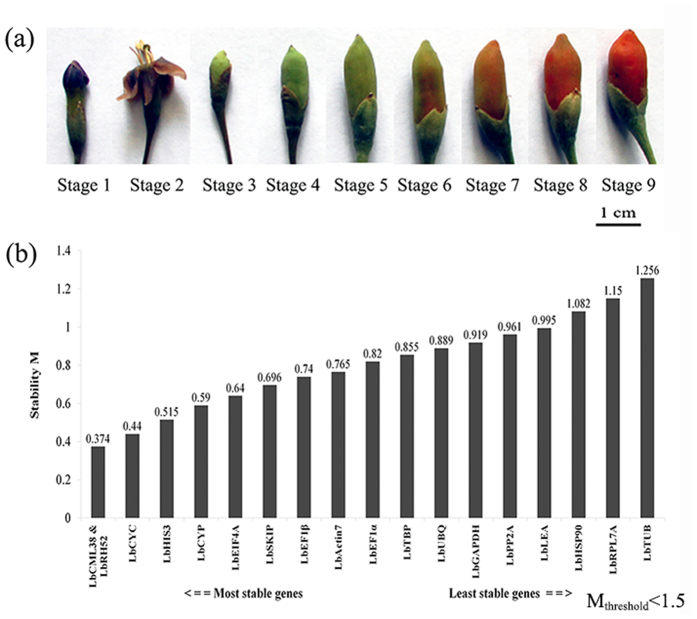
Sampling of materials and ranking of candidate reference genes. (**a**) Samples representing different developmental stages of fruits for sequencing. (**b**) Comprehensive ranking of candidate reference genes based on their expression stability in all 14 samples calculated by geNorm. Stage 2 represents the flowering stage. Stages 3, 4 and 7 represent samples 18, 31 and 40 days after flowering, respectively. Scale bar = 1 cm. The y-axis represents the expression stability of gene based on normalization M. M_threshold_ < 1.5 for solo calculation.

We used geNorm to compare and rank the M value of each candidate reference gene in terms of expression stability. The higher the M value, the lower the stability, and *vice versa*. The default cutoff value of geNorm software is 1.5^5^. All candidate reference genes showed an M value lower than 1.5 (Fig. 1b). Based on the scores obtained from the 14 samples in different organs, at different developmental stages, and after different treatments, *LbCML38* and *LbRH52* were chosen as the best reference genes with an M value of 0.374. In leaves, *LbCML38* and *LbRH52* showed the best expression stability with an M value of 0.273 (Supplementary Table S3). In fruits and flowers, *LbTBP* demonstrated better stability with an M value of 0.249 and 0.142, respectively. Under drought stress condition, *LbCYC* and *LbCML38* were the most stably expressed genes with an M value of 0.32 (Supplementary Table S2). However, commonly used reference genes such as *LbCYC, LbActin7* and *LbHIS3* showed mediocre expression stability. *LbHSP90, LbTUB* and *LbGAPDH* showed poor expression stability with the lowest ranking among these tested candidate reference genes (Supplementary Tables S2 and S3).

**Figure 2:**
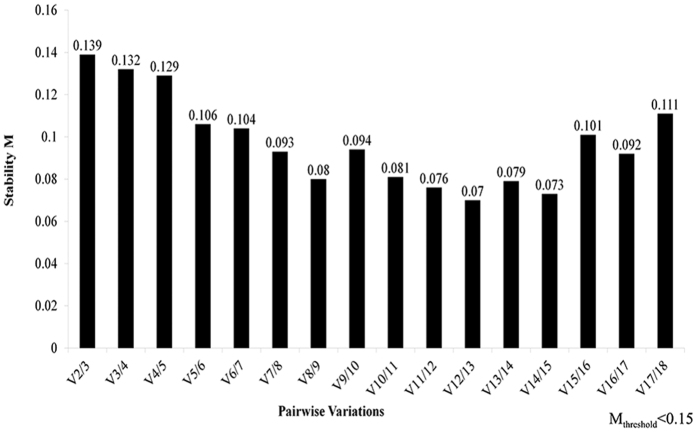
Comprehensive ranking of candidate reference genes based on their expression stability in all 14 samples calculated by geNorm pairwise. The y-axis represents the expression stability of genes based on normalization M. M_threshold_ < 0.15 for pairwise comparison.

We also determined the optimal number of reference genes required under a particular condition by analyzing their paring difference value V_n/n+1_. Typically, the threshold was set to 0.15 to select the best reference gene. When the paired value is lower than 0.15, additional (n + 1) reference genes are not necessary. After combining the analyses of all samples in different organs, at different developmental stages and after drought stress treatment together, V_2/3_ (0.139) was lower than the threshold of 0.15, indicating that the optimal number of reference genes needed was 2 (Fig. 2), and no need to introduce a third reference gene for calibration. The best combination of reference genes was *LbCML38* and *LbRH52*. A combination of *LbCYC* and *LbCML38* is optimal to analyze samples in different organs and at different developmental stages (Supplementary Tables S1, S2 and S3).

### NormFinder analysis

**Figure 3:**
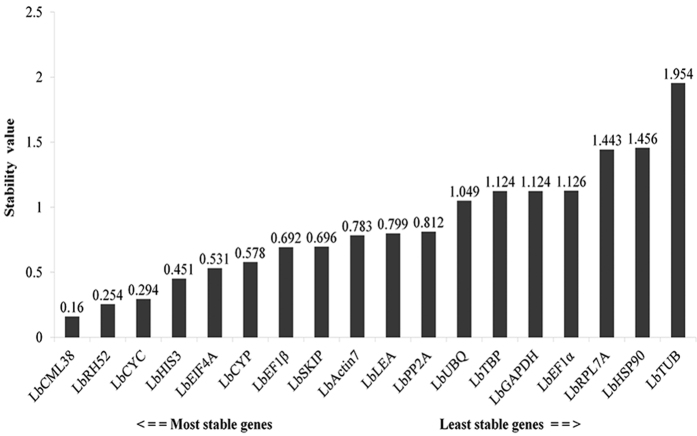
Comprehensive ranking of candidate reference genes based on their expression stability in all 14 samples calculated by NormFinder. The y-axis represents the expression stability of genes based on normalization stability values.

Similar to geNorm, NormFinder evaluates reference genes by calculating their expression stability. Combined analyses of all samples showed that expression of *LbCML38* and *LbRH52* was the most stable with values of 0.16 and 0.254, respectively (Fig. 3). In different organs, *LbRH52* was the most stably expressed reference gene in leaves (0.079), whereas *LbActin7* was appropriate reference gene in fruits and flowers (Supplementary Table S3). Under drought stress condition, *LbCML38* showed the highest expression stability with a value of 0.16 (Supplementary Table S2). Among the commonly used reference genes, NormFinder analyses confirmed the validity of *LbCYC, LbHIS3* and *LbEIF4A*, with a value less than 0.5. On the contrary, *LbHSP90, LbGAPDH* and *LbTUB* showed poor expression stability (Fig. 3, Supplementary Table S3), and were not suitable to be used as reference genes.

### BestKeeper analysis

**Figure 4:**
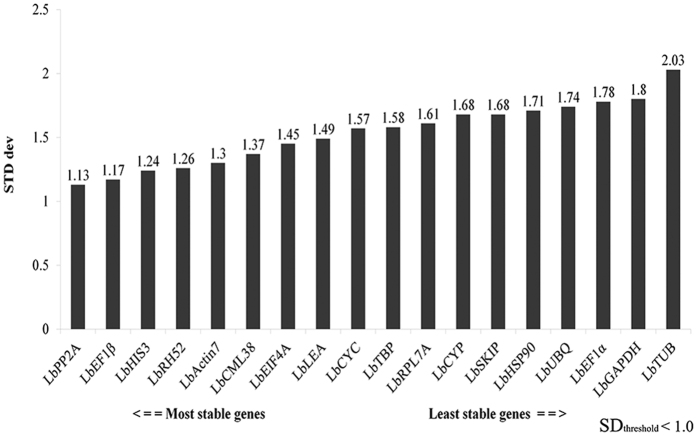
Comprehensive ranking of candidate reference genes based on their expression stability in all 14 samples calculated by BestKeeper. The y-axis represents the expression stability of genes based on STD dev values. SD_threshold_ < 1.0 for BestKeeper analysis.

Results from BestKeeper analysis is slightly different from that of geNorm and NormFinder analyses. When combining the analyses of all samples in different organs, at different developmental stages and under drought stress condition together, *LbPP2A* (SD = 1.13) was identified as the best reference gene (Fig. 4). *LbPP2A* was the most stably expressed gene in leaves (SD = 0.85). Whereas *LbEF1β* (SD = 0.77) and *LbHSP90* (SD = 0.11) were respectively selected as the best reference gene in fruits and flowers (Supplementary Table S3). *LbGAPDH* (SD = 1.8) and *LbTUB* (SD = 2.03) were the worst candidate reference genes. Under drought stress condition, *LbHIS3* (SD = 0.98) was the most stably expressed reference gene (Supplementary Table S2).

### RefFinder analysis

**Figure 5:**
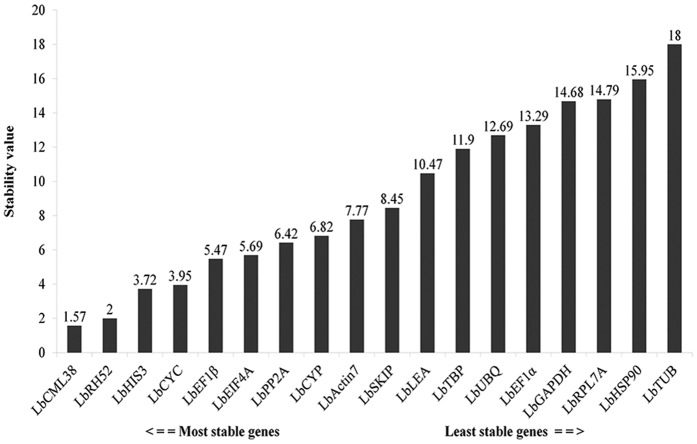
Comprehensive ranking of candidate reference genes based on their expression stability in all 14 samples calculated by RefFinder. The y-axis represents the expression stability of genes based on normalization stability values.

Statistical analyses showed that the stability value ranged from 1.57 to 18 among the 18 selected candidate reference genes in the combined analyses of all samples in different organs, at different developmental stages and under drought stress condition evaluated by RefFinder (Fig. 5). *LbCML38* and *LbRH52* were identified as the most stably expressed two reference genes with an average value less than 2. *LbGAPDH, LbHSP90* and *LbTUB* were the worst. In addition, RefFinder ranked *LbCML38, LbRH52, LbHIS3* and *LbCYC* as the top four reference genes, which was consistent with the rankings obtained with geNorm and NormFinder. Collective evidence suggested that *LbCML38* and *LbRH52* were the best reference genes under the tested conditions. Individual factor analysis indicated that *LbCML32* was the best reference gene under drought stress condition (Supplementary Table S2). *LbRH52* and *LbActin7* were identified as the best reference genes for target gene calibration in leaves, fruits and flowers (Supplementary Table S3).

**Figure 6:**
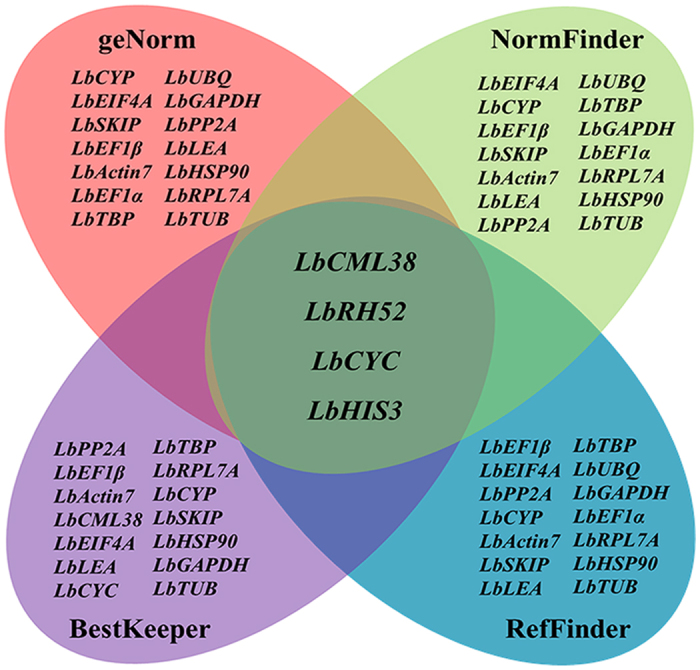
Venn diagram showing the most stable reference genes identified by the geNorm, NormFinder, BestKeeper and RefFinder algorithms. The intersection part shows the most stable genes in common, especifically, *LbCML38, LbRH52, LbCYC* and *LbHIS3*. Mapping data were derived from Figs 1b, 3, 4 and 5.

**Figure 7:**
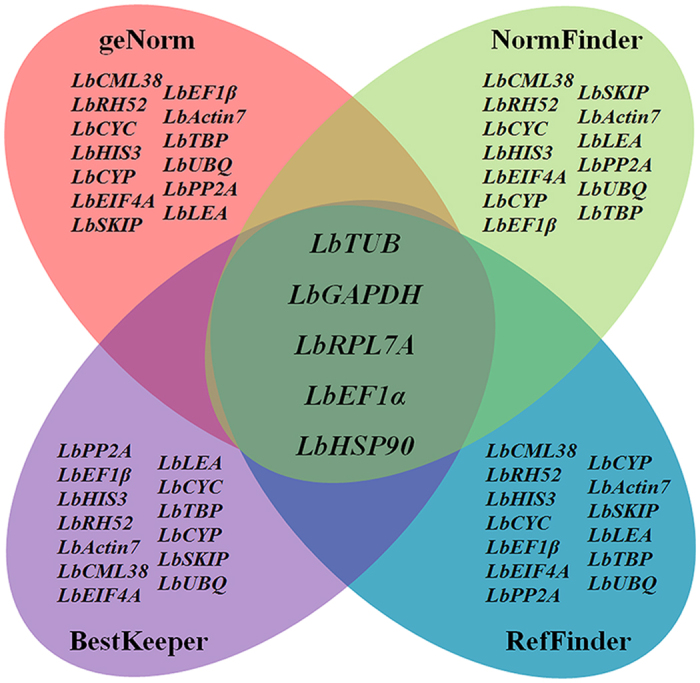
Venn diagram showing the most unstable reference genes identified by the geNorm, NormFinder, BestKeeper and RefFinder algorithms. The intersection part shows the most unstable genes in common, especifically, *LbTUB, LbEF1*α, *LbGAPDH, LbRPL7A* and *LbHSP90*. Mapping data were derived from Figs 1b, 3, 4 and 5.

In summary, these four software algorithms yielded various results in selecting the best reference genes in different organs, at different developmental stages, and under different treatment conditions in Goji. *LbCML38* and *LbRH52* showed relatively stable expression (Fig. 6), whereas *LbTUB, LbHSP90* and *LbGAPDH* were not so stable (Fig. 7). Specifically, *LbActin7* should be carefully used for calibrating gene expression in fruits and flowers. *LbCML38*, especially combination of *LbCML38* and *LbRH52* performed the best stability under most conditions.

### Target gene expression analyses

**Figure 8:**
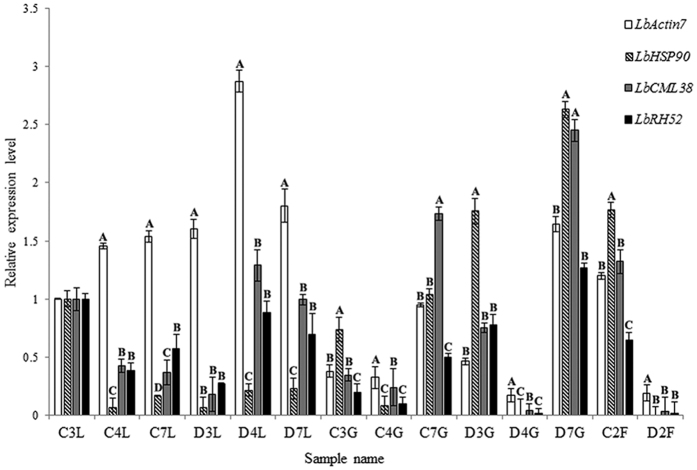
Expression profiles of *LbMYB1* in 14 samples of *L. barbarum* determined by qRT-PCR using *LbAcitn7, LbHSP90, LbCML38* and *LbRH52* as reference genes. Data were compared and analyzed with analyses of variance (ANOVA) and multiple comparisons using the statistical analysis software of SPSS 21 (*P* < 0.01). Error bars show the mean standard error based on triplicates. Letters and numbers in the abscissa axis stand for: C, control group; D, drought stress treatment group; L, leaf; G, Gouji berry; F, flower; 3, development stage 3 (18 days after flowering); 4, development stage 4 (31 days after flowering); 7, development stage 7 (40 days after flowering).

To further re-evaluate the validity of selected reference genes, the expression level of *LbMYB1* in 14 samples was normalized with *LbCML38, LbRH52, LbActin7* and *LbHSP90*. As illustrated in Fig. 8, no significant difference between *LbCML38* and *LbRH52* was observed in most of the treatments when they were used for normalization of *LbMYB1 (P* < 0.01). However, compared with *LbCML38, LbActin7* yielded a higher normalization value of *LbMYB1* in leaves and lower values in fruits and flowers after some specific treatments (*P* < 0.01). *LbMYB1* normalization against *LbHSP90* resulted in higher values in most cases (*P* < 0.01). These results demonstrate that *LbActin7* and *LbHSP90* introduced errors when they were used as reference genes. Therefore, it is crucial to select appropriate reference gene(s) for evaluation of gene expression.

## Discussion

The acreage of Goji in Ningxia and Qianghai provinces of China is growing rapidly due to its economical and medicinal value. However, the molecular mechanism of fruit development, pharmacologically active ingredient accumulation, and stress resistance of it is largely unknown. Screening and selection of stable reference genes for gene expression study in Goji will provide a foundation for elucidating the molecular mechanism. In the absence of systematic profiling of reference genes, we screened the reference genes suitable for samples in different organs, at developmental stages and under drought stress condition of Goji using transcriptomic sequence database for the first time.

Several studies on the screening of reference genes in Solanaceae family plants have been reported. In pepper, *EF1α* and *UEP* were found to be the most stably expressed genes in roots, stems, leaves and flowers under different treatment conditions (salicylic acid, gibberellic acid, cold, heat, salt, and drought)^21^. *EF1α* and *APRT* were the most stably expressed reference genes among 8 commonly used ones when potato plants were exposed to salt and drought stress, respectively^22^,^23^. In tomato (*Solanum lycopersicum*, cv. Suzanne), *RPL2* and *PP2Acs* exhibited as stable expression as *ACT* and *UBI* under nitrogen deficiency, low temperature and different light conditions^24^. Gantasala and co-workers^25^ investigated 6 commonly used reference genes (*18sRNA, APRT, GAPDH, CYP, Actin* and *RuBP*) in egg plants (*Solanum melongena*) and found that *18sRNA, CYP* and *APRT* had the best expression stability.

From the results discussed above, *EF1α* or *Actin* showed relative stable expression in some of the Solanaceae family plants. Liu *et al*. used *Actin* as a reference gene in fruits of two goji cultivars (*Lycium barbarum* L. and *L. ruthenicum* Murr.) for the profiling of genes involved in carotenoid biosynthesis and metabolism^13^. However, our study demonstrated that *LbActin7* exhibited moderately stable expression in some of the treatments in flowers and fruits, but introduced significant errors when normalizing the target gene expression level in leaves and fruits at specific developmental stages (Fig. 8). These results suggest that special caution should be paid when using *LbActin7* as a reference gene. Besides, commonly used *LbGAPDH, LbHSP90*, and especially *LbTUB*, were not suitable to be used as reference genes in Goji (Figs 6 and 7). This was further confirmed in the normalization of *LbMYB1* gene expression (Fig. 8). We also tested the expression stability of established reference genes in Solanaceae family plants including *LbRPL, LbPP2A* and *LbEF1α*. Our data suggest that they are not suitable to be used as reference genes in all the tested samples of Goji (Figs 1b, 2, 3, 4, 5 and Supplementary Tables S1, S2 and S3). The expression stability of *GAPDH, EF1α, Actin* and other reference genes has been questioned in some reports^6^,^26^,^27^,^28^. The discrepancies between these results tested in Solanaceae materials and our data could be due to the different genetic backgrounds of plant species as well as the different treatment conditions. Therefore, selection of appropriate reference genes is critical in the genomic function study of Goji or a comparative study of different plant lines in the Solanaceae family.

DNA chips and next-generation sequencing provide a novel approach for reference gene screening of non-model organisms that lack whole genome information. Czechowski and co-workers firstly proposed to screen reference genes using *Arabidopsis* whole genome Affymetrix ATH1 chips^17^. González-Agüero *et al*. further summarized and refined the analytic process for reference gene screening from RNA-Seq data in grape^20^. A total number of 19 candidate reference genes were identified from 242 non-differentially expressed genes (NDE) using CV < 0.4 of the total read as the screening threshold. qRT-PCR results showed that *VvAIG1* and *VvTCPB* were the most stably expressed reference genes in 14 grape lines, at 4 developmental stages, and under gibberellic acid treatment condition. Similarly, studies with oil-tea camellia^29^, *Striga hermonthica*^30^ and plums^16^ corroborated the approach of using RNA-Seq database to screen reference genes. In this study, we adopted even stricter screening threshold (CV < 0.3) to select 10 functional genes from 1272 raw fragments according to the analytic protocol proposed previously^20^, and three classical reference genes *LbEIF4A, LbUBQ* and *LbEF1α* were included with a CV value less than 0.1 (Table 1). They showed better stability than that of *LbTUB, LbHSP90* and *LbGAPDH* as confirmed by qRT-PCR (Table 2), all of which had higher CV values of raw fragments and C_T_ values.

Taken together, our studies confirmed the correlation between the transcriptional expression stability and qRT-PCR results, thus it is appropriate to select the transcripts with smaller CV values as candidate reference genes during preliminary screening with sequencing data. Collective evidence suggests that *LbCML38* and *LbRH52* could be used as the best reference genes for gene expression study in Goji.

## Methods

### Sample preparation and treatment

Ningxia goji (*Lycium barbarum* L.) Ningqi I was cultivated in field and collected. Plants grown under normal condition were used as control (control, C). Drought stress treatment was performed as described previously^31^ with minor modification: four-year-old adult plants were transplanted into pots filled with a mixture of fertilizer, sand and loam (1:1:3 v/v). The maximal water holding capacity of soil in field was measured and determined as 18%. Transplanted plants at vegetative growth stage with similar physiological state were selected for drought stress treatment. During the drought stress period, water holding capacity of soil in pots was maintained at 40% to 45% of the maximal field level. The water capacity of soil was measured at a fixed time point every day using soil moisture meter TDR300 (Spectrum, USA), and maintained at a specific level by artificial replenishment. Ten plants were individually cultivated in pots for each treatment. Sequencing samples including equal amounts of leaves (L), flowers (F) and fruits (G) from control and drought-treated plants were collected 18 (developmental stage 3), 31 (developmental stage 4) and 40 (developmental stage 7) days after flowering (Fig. 1a). Control leaves were collected from the same plants at developmental stage 3.

### Total RNA isolation and sequencing database establishment

Total RNA was extracted using plant total RNA extraction kit (Tiangen Biotechnology, PRC). Genomic DNA was eliminated by treating each sample with RNase-free DNase I (TAKARA BIO INC., code 2270A) according to the instruction manual. The purity of total RNA extracted was checked using a NanoDrop 2000 spectrophotometer. Samples with an absorbance ratio at OD260/280 between 2.0 and 2.2 were used for further analyses. The concentration and quality of extracted RNA was determined using Agilent 2000 bioanalyzer (Agilent, USA). First-strand cDNA was synthesized from magnetic beads-enriched poly(A)-mRNA using random hexamers, followed by buffer addition to synthesize the complement strand. Synthesized cDNA was purified using Qiaquick PCR purification kit (Qiagen, USA) and eluted with EB elution buffer. Purified cDNA was mixed with poly(A) tail and sequencing adaptors. Appropriately sized cDNA fragments were selected by agarose electrophoresis and amplified by PCR. The library was sequenced on Illumina HiSeq2000 using *de novo* PE 100 sequencing strategy.

### Sequence assembly and annotation of basic bioinformatics

The original imaging data obtained from sequencing were processed into raw reads by base calling, followed by filtration of noises and low quality data to obtain clean reads. The *de novo* assembler program Trinity was used to assemble the short reads into contigs, scaffolds and Unigene, respectively^32^. Unigene sequences were blasted against that of Nr, SwissProt, GO, KEGG and COG databases (E-value < 0.00001) to obtain homologous proteins with high sequence similarity to the reference protein. When Unigene sequences failed to match sequences in the databases, ESTScan software was used to predict the coding region and sequence orientation^33^. Unigene expression level was calculated using FPKM method (Fragments Per kb per Million fragments)^34^. The RNA-Seq data used in this study is available at the Sequence Read Archive (SRA, http://www.ncbi.nlm.nih.gov/sra) of the National Center for Biotechnology Information (NCBI) with an accession number SRP063577.

### Reference gene screening and primer design

Two approaches were used to select reference genes: 1) Excavation of classical reference genes: 8 classical reference genes including *CYP, TBP, GAPDH, TUB, EF1α, Actin7, HSP90* and *HIS3* derived from tomato or *Arabidopsis* were used as template to screen homologous genes from prepared RNA-Seq database of *L. barbarum*; 2) Based on the method described by González-Agüero *et al*.^20^, 10 stably expressed Unigenes (*LbCYC, LbEIF, LbPP2A, LbUBQ, LbCML38, LbLEA, LbEF1β, LbSKIP, LbRH52* and *LbRPL7A*) were selected out of 1272 transcripts (raw fragments > 500, and the coefficient of variation < 0.3). Primers were designed using Primer 3 software (http://frodo.wi.mit.edu/primer3/). Data information about the candidate genes and primers are listed in Tables 1 and 2.

### qRT-PCR and data analysis

**S**ynthesis of cDNA was performed with 0.5–1 μL total RNA (the final content of RNA in the reaction mixture was adjusted to 1 μg for all samples) according to the instruction manual of the cDNA synthesis System (TRANSGEN BIOTECH INC., code AU311–02) in a total volume of 20 μL. Quantitative RT-PCR analysis of the cDNA of 14 samples was performed using StepOne Real-time PCR Systems (Applied Biosystems, USA). A total amount of 20 μl of PCR reaction mix containing 10 μl of Power SYBR Green PCR Master Mix, 5 μl of diluted cDNA, 0.5 μl (10 pmol) each of forward and reverse primers, and ddH_2_O was prepared. The thermocycling condition was set as follows: initial denaturation at 95 °C for 10 mins, 40 cycles of denaturation at 95 °C for 15 seconds, annealing at 60–62 °C for 15 seconds, and extension at 72 °C for 25 seconds. Fluorescent signals were collected after each cycle. Each sample was run in triplicate along with a negative control. Amplified products were checked on 1% agarose gel.

The first-strand cDNA was synthesized after six rounds using a five-fold serial dilution. Each PCR reaction was conducted in triplicate using diluted cDNA as template. The C_T_ values of samples were generated automatically after qRT-PCR. A standard curve was also generated and the melting curve was analyzed to determine the specificity of PCR products. The amplification efficiency (E) of each candidate reference gene was calculated with the slope of the standard curve according to the equation E = (10^−1^/slope − 1) × 100%^17^.

The stability of candidate reference genes was evaluated by geNorm, NormFinder, BestKeeper and RefFinder using obtained C_T_ values of the samples. The average expression stability value (M) of each candidate reference gene was calculated by geNorm^5^. The higher the M value, the less stable the gene expression was, and *vice versa*. Meanwhile, paired difference analysis (V_n/n+1_) of candidate reference gene normalization factor was used to determine the optimal number of required reference genes. NormFinder was used to determine the candidate reference gene stability using the combined variances within and between groups^35^. The lower the stability value, the more stable the reference gene expression was, and *vice versa*. BestKeeper was used to determine the candidate reference genes’ standard deviation (SD) and coefficient of variation (CV)^36^. Reference gene with the smallest CV ± SD value was considered the most stably expressed one. All reference genes with a SD value less than 1 were established as stably expressed genes. The smaller the SD value, the more stable the reference gene expression was, and *vice versa*. To calculate the geometric mean values and the ranking of each candidate reference gene, results respectively obtained with geNorm, NormFinder, BestKeeper and Delta C_T_ were integrated using RefFinder (http://www.leonxie.com/referencegene.php). The lower the ranking index, the more stable the reference gene expression was, and *vice versa*.

### Reference gene validation

For confirmation of selected reference gene validity, *LbMYB1* which encodes a MYB transcription factor involved in drought stress response and flavonoid anabolism was selected as a target gene^37^. The expression levels of *LbMYB1* in difference samples were normalized against those of the most stable reference genes *LbCML38* and *LbRH52*, the moderate stable reference gene *LbActin7*, and the less stable reference gene *LbHSP90*, respectively. Data were compared and analyzed with analysis of variance (ANOVA) and multiple comparisons using the statistical analysis software of SPSS 21. Values of *P* < 0.01 were considered statistically significant difference.

## Additional Information

**How to cite this article**: Gong, L. *et al. LbCML38* and *LbRH52*, two reference genes derived from RNA-Seq data suitable for assessing gene expression in *Lycium barbarum* L. *Sci. Rep.* **6**, 37031; doi: 10.1038/srep37031 (2016).

**Publisher’s note:** Springer Nature remains neutral with regard to jurisdictional claims in published maps and institutional affiliations.

## Supplementary Material

Supplementary Information

Supplementary Table S1

Supplementary Table S2

Supplementary Table S3

## Figures and Tables

**Table 1:** Characteristics of candidate reference genes.

Gene ID	Gene abbreviation	Mean raw fragments	Standard Deviation (SD)	Coefficient of variation (CV)	Swissprot annotation
CL10201.Contig1_All	*LbCYP*	1142.214286	718.6318388	0.62915676	Peptidyl-prolyl cis-trans isomerase CYP20-2, chloroplastic OS = *Arabidopsis thaliana* GN = CYP20-2 PE = 1 SV = 1
CL1082.Contig3_All	*LbTBP*	138.5714286	38.5141618	0.27793725	TATA-box-binding protein OS = *Solanum tuberosum* GN = TBP PE = 2 SV = 1
Unigene36249_All	*LbGAPDH*	145.4285714	68.28527748	0.46954513	Glyceraldehyde-3-phosphate dehydrogenase 1, cytosolic OS = *Arabidopsis thaliana* GN = GAPC1 PE = 1 SV = 2
CL13903.Contig10_All	*LbTUB*	57.57142857	57.88412069	1.005431377	Tubulin beta-2 chain OS = *Solanum tuberosum* GN = TUBB2 PE = 2 SV = 1
CL2539.Contig4_All	*LbEF1α*	3196.071429	823.6067456	0.257693473	Elongation factor 1-alpha OS = *Solanum lycopersicum* PE = 2 SV = 1
CL4826.Contig3_All	*LbActin7*	626.5714286	191.7922411	0.306097968	Actin-7 OS = *Arabidopsis thaliana* GN = ACT7 PE = 1 SV = 1
Unigene44516_All	*LbHSP90*	126.0714286	53.97501457	0.428130427	Heat shock protein 90–2 OS = *Arabidopsis thaliana* GN = HSP90-2 PE = 1 SV = 1
CL13810.Contig1_All	*LbHIS3*	2307.928571	612.946536	0.265582975	Histone H3.3 OS = *Vitis vinifera* PE = 2 SV = 3
CL10058.Contig1_All	*LbCYC*	1009	92.22297394	0.091400371	Cyclin-B1-5 OS = *Arabidopsis thaliana* GN = CYCB1-5 PE = 2 SV = 3
Unigene55248_All	*LbEIF4A*	2087.928571	165.8161467	0.07941658	Eukaryotic initiation factor 4A-3 OS = *Nicotiana plumbaginifolia* PE = 2 SV = 1
Unigene19346_All	*LbPP2A*	831.8571429	99.63923937	0.119779268	Serine/threonine-protein phosphatase PP2A catalytic subunit OS = *Nicotiana tabacum* PE = 2 SV = 1
CL11175.Contig21_All	*LbUBQ*	1211.428571	186.5371542	0.153981141	Polyubiquitin 10 OS = *Arabidopsis thaliana* GN = UBQ10 PE = 1 SV = 2
Unigene15131_All	*LbCML38*	695	106.1066227	0.1526714	Calcium-binding protein CML38 OS = *Arabidopsis thaliana* GN = CML38 PE = 2 SV = 1
CL7969.Contig1_All	*LbLEA*	525.7142857	113.3995174	0.215705604	Late embryogenesis abundant protein Lea14-A OS = *Gossypium hirsutum* GN = LEA14-A PE = 2 SV = 1
Unigene31075_All	*LbEF1β*	5663	1138.411849	0.201026285	Elongation factor 1-beta OS = *Oryza sativa* subsp. *japonica* GN = Os07g0662500 PE = 1 SV = 3
Unigene26461_All	*LbSKIP*	612.5	120.2361459	0.196303912	F-box protein SKIP31 OS = *Arabidopsis thaliana* GN = SKIP31 PE = 1 SV = 1
Unigene55138_All	*LbRH52*	950.4285714	113.8816621	0.119821379	DEAD-box ATP-dependent RNA helicase 52 OS = *Arabidopsis thaliana* GN = RH52 PE = 2 SV = 1
Unigene59903_All	*LbRPL7A*	793.6428571	149.1166849	0.187888902	60S ribosomal protein L7-1 OS = *Arabidopsis thaliana* GN = RPL7A PE = 2 SV = 1
Unigene59939_All	*LbMYB1*	2982.571429	697.3143085	0.233796348	Transcription factor MYB1R1 OS = *Solanum tuberosum* PE = 2 SV = 1

**Table 2:** Primer sequences and amplicon characteristics of the 18 reference genes.

Gene abbreviation	Forward primer (5′-3′)	Reverse primer (5′-3′)	TM (°C)	Amplicon size (nt)	PCR efficiency (%)	Correlation coefficient (R^2^)	Mean CT	Standard Deviation (SD)	Coefficient of variation (CV)
*LbCYP*	TCGTTGCGTCTGGCTACTTCA	CTGTCTGCGGCACATCATCAC	61	208	91.3	0.9953	20.19	2.166658889	0.107307139
*LbTBP*	CGACGAATGGCAGATCAAGGATA	CAAGTTCACCGTTGAGACAATGTT	62	108	101.0	0.9937	24.67	2.140584019	0.086744429
*LbGAPDH*	CACGGTCAATGGAAGCACAAT	GCAGCAGCCTTGTCTTTATCC	60	179	94.8	0.9775	22.46	2.267361358	0.100925407
*LbTUB*	GTCCAGAACAAGAACTCGTCCT	CGCCCTCCTCATCATACTCCT	60	325	96.1	0.9747	23.58	2.294896689	0.097316984
*LbEF1α*	TCGTGTGGAGACTGGTGTAATC	TCGCCTGTCAATCTTGGTCAA	60	352	92.2	0.9973	17.43	2.401203192	0.137725035
*LbActin7*	GGTCCTCTTCCAGCCATCCAT	TGAGCCACCACTGAGCACAA	62	133	90.6	0.9962	20.29	1.841419014	0.090747549
*LbHSP90*	TCCTGATAGTCCTGCTGAGTTGG	TTCCGTTGATGCTTCTGCTGATG	60	121	97.3	0.9502	18.55	2.043184756	0.110139076
*LbHIS3*	CACTACAGGTGGTGTGAAGAAG	CACGAACAAGCCTCTGGAAG	61	128	91.9	0.9957	17.18	1.561123661	0.090835934
*LbCYC*	TTCTTGTCATACTCGGTGTTGTGT	CTGAAGTTGTCTCTGTGCCTGTA	60	119	98.5	0.9977	21.01	1.875417615	0.089258037
*LbEIF4A*	ACGGAGATATGCCACAGAAGGAG	AGAGCGACCAATGCGATGAATG	60	182	92.2	0.9543	20.79	1.830072065	0.087990277
*LbPP2A*	GAGATGCTGTGAAGAGATGGTGAA	AGAAGATTACACGAACGCTCATTGA	61	246	105.0	0.9857	21.17	1.30967704	0.061843202
*LbUBQ*	GGCATTCCTCCAGATCAGCAA	GTGTCCGAACTCTCAACCTCAA	60	291	106.1	0.9993	20.14	2.090803559	0.103768094
*LbCML38*	CGGTGGTTCTTCTGGTTCATCAA	TCTTCTTGTGCCTCCTCATTAACTC	62	192	105.7	0.9958	21.68	1.708817146	0.078819113
*LbLEA*	TGTCTGGCTCTCCGATGTCAA	TCTCCAACTTCAATCCTCATCTTCA	61	310	92.6	0.9851	18.37	1.741954661	0.094816225
*LbEF1β*	GACGATGACGACGACGACAT	GCGAACAGCCTCCTCAAGT	60	186	94.9	0.9947	18.88	1.674620435	0.088683577
*LbSKIP*	TGAGGAGGAGGAGGAGGAAGAA	TGAGGATGTGACGGAGCAACT	60	293	102.4	0.9936	25.02	2.10058639	0.083924345
*LbRH52*	GCAGGCAAGTCAGGATTAGCA	CGCATAACGAGTCAACCATTCAG	62	123	97.5	0.9972	22.02	1.636191649	0.074287127
*LbRPL7A*	CCTTTACTCTCACCAAGCCAGAA	TCACAACTACGATACACCGAACA	60	162	110.1	0.9536	20.99	1.774507767	0.084515702
*LbMYB1*	TCCGCCGAAGCAACCTCAAC	CATAGGAACAGGACGAACCAGCAT	60	290	108.2	0.9809	21.11	3.398526812	0.16096409
