# Supplementary material for: LbCML38 and LbRH52, two reference genes derived from RNA-Seq data suitable for assessing gene expression in Lycium barbarum L
Source: Sci Rep. 2016 Nov 14;6:37031. doi: 10.1038/srep37031 (PMC5107986; doi:10.1038/srep37031)
Supplement: Supplementary Information [file srep37031-s1.doc]

*LbCML38* and *LbRH52*, two reference genes derived from RNA-Seq data suitable for assessing gene expression in *Lycium* *barbarum* L.

Lei GONG^1,†^, Yajun YANG^2,†^, Yuchao CHEN^1^, Jing SHI^2^, Yuxia SONG^1,*^ and Hongxia ZHANG^3,4*^

^1^Ningxia Key Laboratory for Agrobiotechnology, Agricultural Bio-Technology Center, Ningxia Academy of Agriculture and Forestry Science, 590 Huanghe East Road, Yinchuan, Ningxia Hui Nationality Autonomous Region, China 750002

^2^School of Life Sciences, Ningxia University, 489 Helanshan West Road, Yinchuan, Ningxia Hui Nationality Autonomous Region, China 750021

^3^National Key Laboratory of Plant Molecular Genetics, Shanghai Institute of Plant Physiology and Ecology, Chinese Academy of Sciences, 300 Fenglin Road, Shanghai, China 200032

^4^College of Agriculture, Ludong University, 186 Hongqizhong Road, Yantai, China 264025

^*^Corresponding: hxzhang@sippe.ac.cn, songyx666@163.com

^†^These authors contributed equally to this work.

**Supplementary information:**

Figure S1. Agarose gel electrophoresis analyses of 18 candidate reference genes. Unique amplicons of expected lengths were detected without primer dimmers and non-specific products.


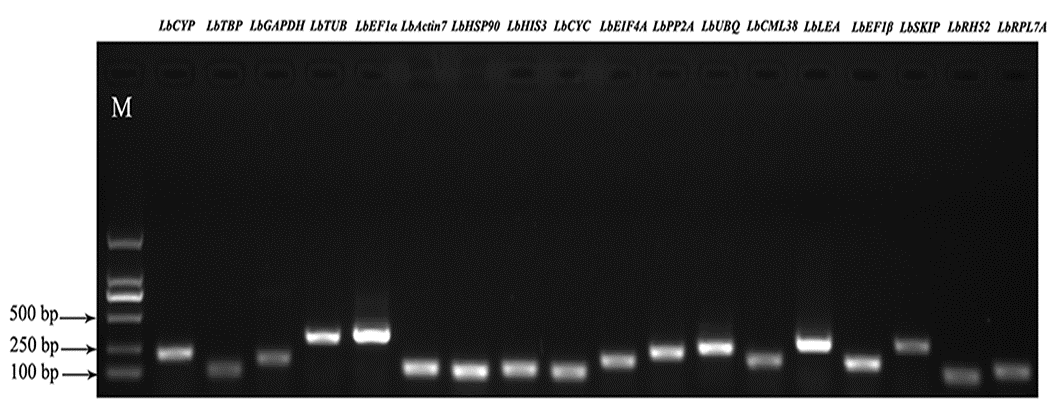


Table S1. Expression stability values and ranking order of 18 reference genes sorted by developmental stage with four different statistical algorithms.

Table S2. Expression stability values and ranking order of 18 reference genes sorted by treatment condition with four different statistical algorithms.

Table S3. Ranking order of 18 reference genes sorted by different organs with four different statistical algorithms.
